# Compound and heterozygous mutations of *KCNQ1* in long QT syndrome with familial history of unexplained sudden death: Identified by analysis of whole exome sequencing and predisposing genes

**DOI:** 10.1111/anec.12694

**Published:** 2019-09-29

**Authors:** Yubi Lin, Ting Zhao, Siqi He, Jiana Huang, Qianru Liu, Zhe Yang, Jiading Qin, Nan Yu, Hongyun Lu, Xiufang Lin

**Affiliations:** ^1^ Department of Cardiology and Cardiovascular Intervention Interventional Medical Center The Fifth Affiliated Hospital of Sun Yat-sen University Zhuhai China; ^2^ Guangdong Provincial Key Laboratory of Biomedical Imaging and Guangdong Provincial Engineering Research Center of Molecular Imaging The Fifth Affiliated Hospital, Sun Yat-sen University Zhuhai China; ^3^ Guangdong Provincial People's Hospital, Guangdong Academy of Medical Sciences, Guangdong Cardiovascular Institute, Guangdong Geriatrics Institute Guangzhou China; ^4^ The Sixth Affiliated Hospital of Sun Yat-sen University Guangzhou China; ^5^ Jinan University Guangzhou China; ^6^ Department of Clinical Laboratory Zhujiang Hospital of Southern Medical University Guangzhou China; ^7^ Department of Endocrinology The Fifth Affiliated Hospital of Sun Yat-sen University Zhuhai China

**Keywords:** genetics, KCNQ1, long QT syndrome, sudden death

## Abstract

**Introduction:**

Long QT syndrome (LQTS) increases the risk of life‐threatening arrhythmia in young individuals with structurally normal hearts. Sixteen genes such as the KCNQ1, KCNH2, and SCN5A have been reported for association with LQTS.

**Case presentation:**

We identified the compound heterozygous mutations in the *KCNQ1* gene at c. G527A (p.W176X) and c.G1765A (p.G589S) predicted as “damaging.” The in‐silico analysis showed that when compared to the characteristics of mRNA and protein of wild‐type KCNQ1, the mRNA of c.G527A mutation was significantly different in the centroid secondary structure. The subunit coded by W176X would lose the transmembrane domains S3–S6 and helices A‐D. The protein secondary structure of G589S was slightly shortened in helix structure; the protein physics‐chemical parameters of W176X and G589S significantly and slightly changed, respectively.

**Conclusions:**

The compound heterozygous mutations of W176X and G589S coexisting in *KCNQ1* gene of homologous chromosomes, resulting in more severe phenotype, are the likely pathogenic and genetic risks of LQTS and USD in this Chinese family.

**Established Facts**

- Long QT syndrome (LQTS) increases the risk of life‐threatening arrhythmia in young individuals with structurally normal hearts.
- Mutations in KCNQ1 causing loss‐of‐function and gain‐of‐function can lead to type 1 LQTS, AF, and even fatal arrhythmia.
- Some limited reports revealed that compound heterozygous mutations in KCNQ1 genes aggravated the phenotype of LQTS.

**Novel Insights**

- In our case report, the compound heterozygous mutations of W176X and G589S in KCNQ1 gene were identified in the patient with type 1 LQTs and familial history of unexplained sudden death (USD). The coexisting interaction of both mutations, resulting in more severe phenotype, may be the important pathogenic and genetic risks for LQTS and USD.

## 1 INTRODUCTION

Long QT syndrome (LQTS) increases the risk of life‐threatening arrhythmia (e.g., torsade de pointes), which leads to syncope, seizures, and unexplained sudden death (USD) in young individuals with structurally normal hearts. LQTS is typically inherited as an autosomal dominant trait while recessive inheritance is observed in rare cases. Recessive inherited LQTS is characterized by severe cardiac phenotype and multisystem syndrome disorders, such as Ankyrin B syndrome, Andersen‐Tawil syndrome, and Timothy syndrome. So far, 16 genes have been reported for association with LQTS, the KCNQ1, KCNH2, and SCN5A are the most common (Nakano & Shimizu, 2016). Here we found a young member of the Chinese Han family characterized as type 1 LQTS and USD. This family was investigated for potential genetic risk by performing the Whole Exome sequencing (WES) and screening candidate genes related to arrhythmia and cardiomyopathies (Lin et al., 2017).

## 2 CASE PRESENTATION

The Medical Institutional Review Board and Medical Ethics Committees of the Guangdong Medical Institutional Review Board and Medical Ethics Committees [No.GDREC2016001H(R1)] approved this study and written informed consents were obtained from the family members. We collected the peripheral blood sample of II:4 during hospitalization. The detailed clinical information was obtained, including the history, manifestation, initial symptoms, physical examination, electrocardiograms (ECGs), and echocardiograms of the family.

**Figure 1:**
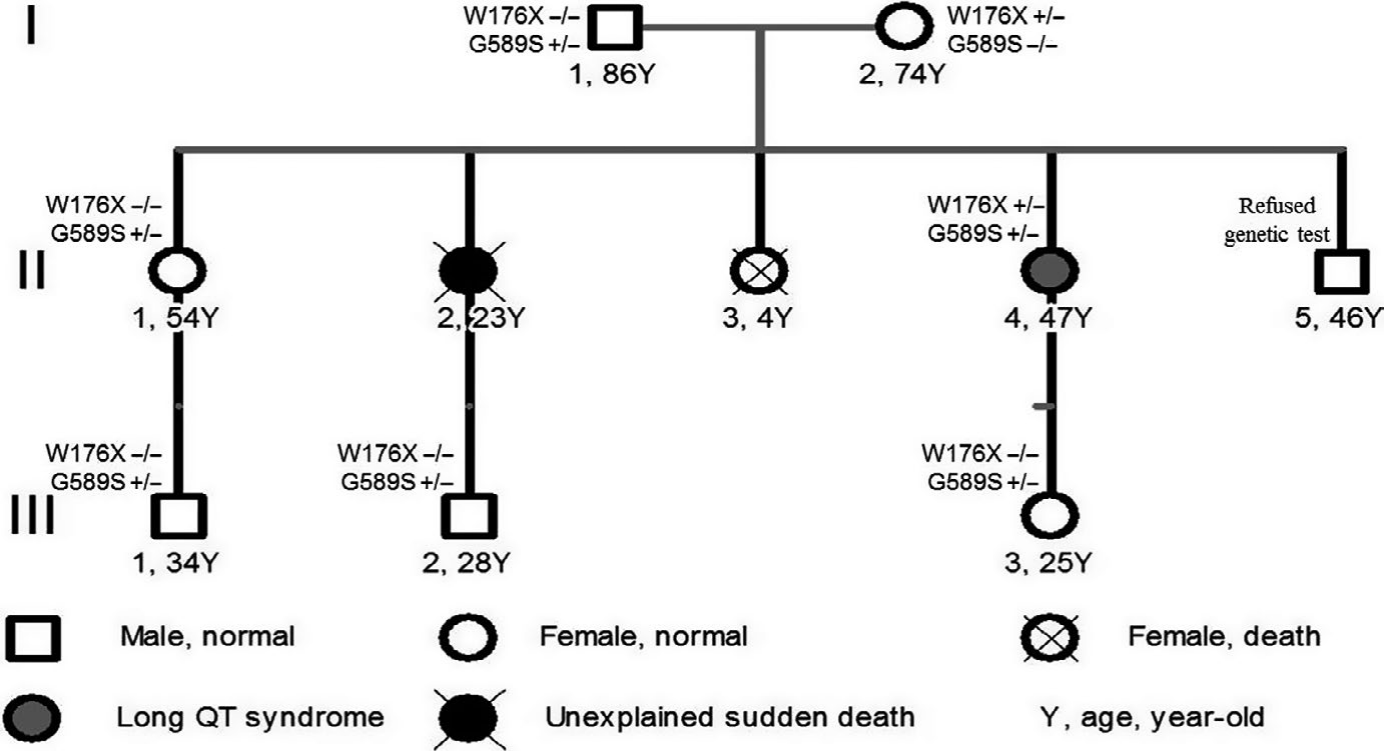
Familial pedigree and phenotype

**Figure 2:**
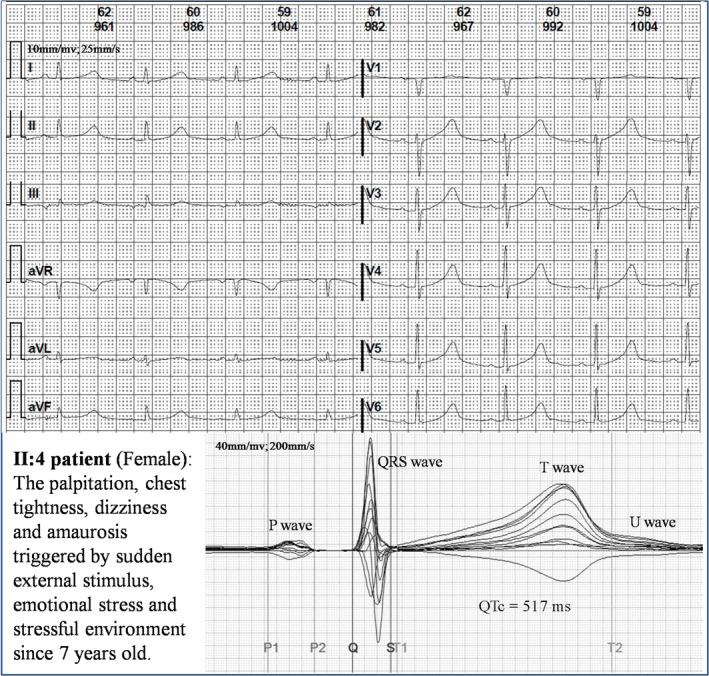
The electrocardiogram of proband II:4

The family pedigree and detailed phenotypes were shown in Figure 1. Once triggered by sudden external stimulus, emotional stress or stressful environment, the proband II: 4 (female, 47 years old) repeatedly presented palpitation, chest tightness, dizziness, and amaurosis since 7 years old. Subsequently, she further developed more symptoms including syncope, urinary and fecal incontinence, and limb weakness, which could be relieved after persisting for approximately two minutes. The electrocardiogram and 24‐hr dynamic electrocardiogram demonstrated the characteristics of type 1 long QT (Figure 2) with QTc 517 milliseconds and occasional ventricular premature. There were no significant abnormalities in the echocardiogram, test of hearing function, computed tomography of the chest, and thyroid examination. Based on the guidelines and the clinical standard score of familial long QT syndrome (Skinner, 2007), the proband II:4 was evaluated with 5.5 points and diagnosed with long QT syndrome. II:4 was first treated by beta‐blocker, but she still repeatedly presented the symptoms while she was suffering from stress. Therefore, she was implanted with an implantable cardioverter defibrillator (ICD) in September 2014 and instructed to avoid as much environmental stress as possible. There was no adverse cardiac event evaluated by ICD monitoring before December 2015. She had no obvious symptom recurrence even without drug therapy during three years of follow‐up. Her sister (II:2) also suffered from similar palpitation, fatigue, amaurosis, and syncope without any medical care and treatment since 10 years old. Consequently, she died of the unexplained sudden death at the age of 23 when she was running. Another sister (II:3) died from the repeated infection at age of 4 years old. I:1 (male, 86 years old) had no clinical symptom or cardiac events associated with arrhythmia before. He had been admitted to countryside hospital for the dyscrasia and marasmus due to frequent severe pulmonary infection. Therefore, we could not record his correct and clear ECG under the turbulence of obvious wheeze. I:2 (female, 74 years old) has no cardiac symptoms with a substantially normal electrocardiogram except for a relatively long QTc (mean QTc per hour 435.86 ± 6.16ms). The other family members do not have cardiac symptoms with normal electrocardiograms.

## 3 METHODS AND RESULTS

### 3.1 WES and predisposing gene analysis

**Figure 3:**
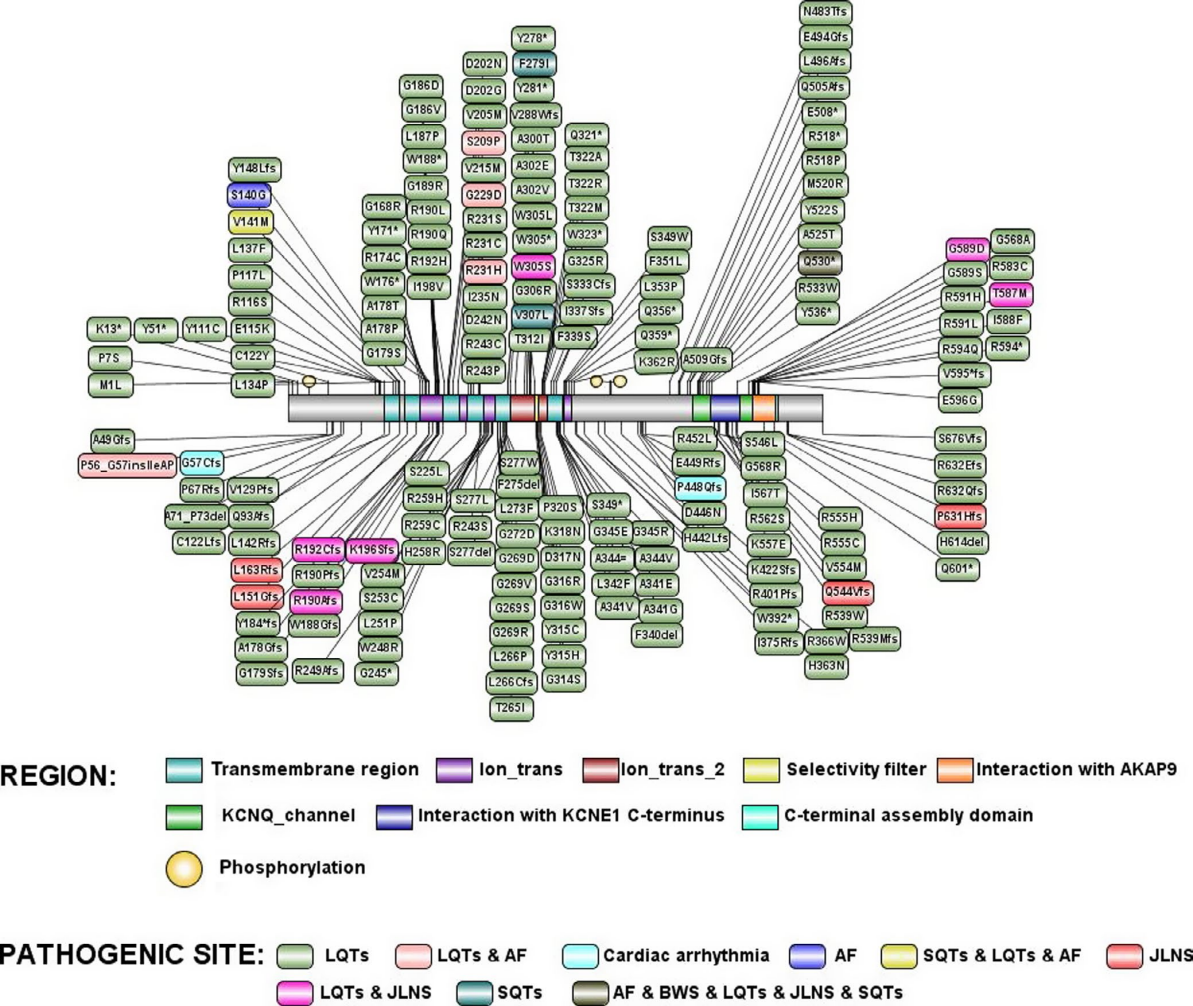
The common pathogenic mutants of KCNQ1 gene in Clinvar database

We conducted the WES or Sanger sequencing of DNA extracted from peripheral blood of the family members except II:2, II:3, and II:5 who had died or refused the genetic test. SNPs and InDels were annotated using a pipeline, in which all insertion and deletion variants occurring at coding regions were considered damaging. Nonsynonymous SNPs were predicted by Sorting Intolerant From Tolerant (SIFT, http://sift.jcvi.org/www/); (Kumar, Henikoff, & Ng, 2009) and PolyPhen‐2 algorithms (Polymorphism Phenotyping v2, http://genetics.bwh.harvard.edu/pph2/);(Adzhubei et al., 2010). Variants in predisposing genes that are associated with hereditary arrhythmias and cardiomyopathies were screened. The filtering criteria are as follows: (a) same variants in the WES data; (b) missense, nonsense, insertion, and deletion variants; (c) SNPs with minor allele frequency not more than 0.01 according to the SNP database of National Center for Biotechnology Information (NCBI) (Smigielski, Sirotkin, Ward, & Sherry, 2000; Via, Gignoux, & Burchard, 2010). We obtained 897 mutations in exon and splicing regions, including 8 mutations of genes predisposing to arrhythmic and cardiomyopathies (Table 1). In this list of the predisposing genes, the KCNQ1 gene encodes a voltage‐gated potassium channel required for repolarization phase of the cardiac action potential, and of which more than 200 mutants (Figure 3) associates with type 1 LQTS, familial atrial fibrillation (AF), Jervell and Lange‐Nielsen syndrome (JLNS), short QT syndrome (SQTs), and Beckwith‐Wiedemann syndrome (BWS) (Table 2). We acquired one stop‐codon mutation of W176X and nonsynonymous mutation of G589S in KCNQ1 gene of homologous chromosomes, carried by II:4 as heterozygous patterns, respectively, which were not existing in the population according to the 1,000 genome database. The other family members only carried one mutation of W176X or G589S (Figure 1). The G589S mutation was predicted as "damaging" by the Polyphen2 algorithm.

**Table 1:** Predisposing gene analysis of whole exome sequencing

Chr	Start	Ref‐Alt	Gene	Amino acid change	Reads	1000G	ESP	SIFT	Polyphen2	dbSNP
chr1	17,345,386	G > C	SDHB	NM_003000:exon8:c.C833G:p.A278G	G/C:19,10	–	–	0.009(D)	0.146(B)	–
chr11	2,591,907	G > A	KCNQ1	NM_000218:exon3:c.G527A:p.W176X	G/A:41,61	–	–	.	.	–
chr11	2,799,238	G > A	KCNQ1	NM_000218:exon15:c.G1765A:p.G589S	G/A:72,78	–	–	0.413(T)	0.985(D)	–
chr16	86,612,444	G > A	FOXL1	NM_005250:exon1:c.G115A:p.A39T	G/A:82,66	–	–	0.222(T)	0.001(B)	–
chr18	19,751,992	G > A	GATA6	NM_005257:exon2:c.G887A:p.G296D	G/A:17,18	–	–	0.087(T)	0.133(B)	–
chr2	179,417,650	T > C	TTN	NM_003319:exon163:c.A62782G:p.K20928E	T/C:27,36	–	–	0.115(T)	0.996(D)	–
chr21	18,965,479	C > T	CXADR	NM_001207066:exon8:c.C1027T:p.P343S	C/T:32,33	–	–	0.023(D)	0.307(B)	–
chr4	120,107,248	C > T	MYOZ2	NM_016599:exon6:c.C688T:p.R230W	C/T:72,59	<0.001	<0.001	0.051(T)	0.979(D)	rs372215131

Abbreviations: B, benign; D, damaging; T, tolerated; –, no report; GT, genotype; ±, heterozygous; 1000G, 1,000 Genomes Project database (2015 version); ESP, Esp6500 datbase.

**Table 2:** Functional protein domain and OMIM diseases related to predisposing genes

Chr	Gene	Functional protein domain	OMIM Disease	Expression in heart
chr1	SDHB	Succinate dehydrogenase complex iron sulfur subunit B	ASD	223.70
chr11	KCNQ1	Ion transport domain; voltage‐dependent Potassium channel, C‐terminal	Type 1 LQTs, AF, JLNS, SQTs	6.99
chr16	FOXL1	Transcription factor; DNA‐binding forkhead domain	–	0.42
chr18	GATA6	GATA‐type transcription activator, N‐terminal	TOF, CHD, AVS, ASD	15.40
chr2	TTN	Fibronectin type III; Immunoglobulin subtype	HM, DCM, HCM	14.69
chr21	CXADR	Ig‐like cell adhesion molecule	–	6.60
chr4	MYOZ2	The z‐line of the sarcomere of cardiac and skeletal muscle cells	HCM	462.93

Note

Abbreviations: AF, atrial fibrillation; ASD, atrial septal defect; AVS, atrioventricular septal defect; CHD, congenital heart defects; Chr, chromosome; DCM, dilated cardiomyopathy; HCM, hypertrophic cardiomyopathy; HM, hereditary myopathy; JLNS, Jervell and Lange‐Nielsen syndrome; LQTs, long QT syndrome; SQTs, short QT syndrome; TOF, Tetralogy of Fallot; –, no report.

## 4 HOLTER RECORDINGS

**Figure 4:**
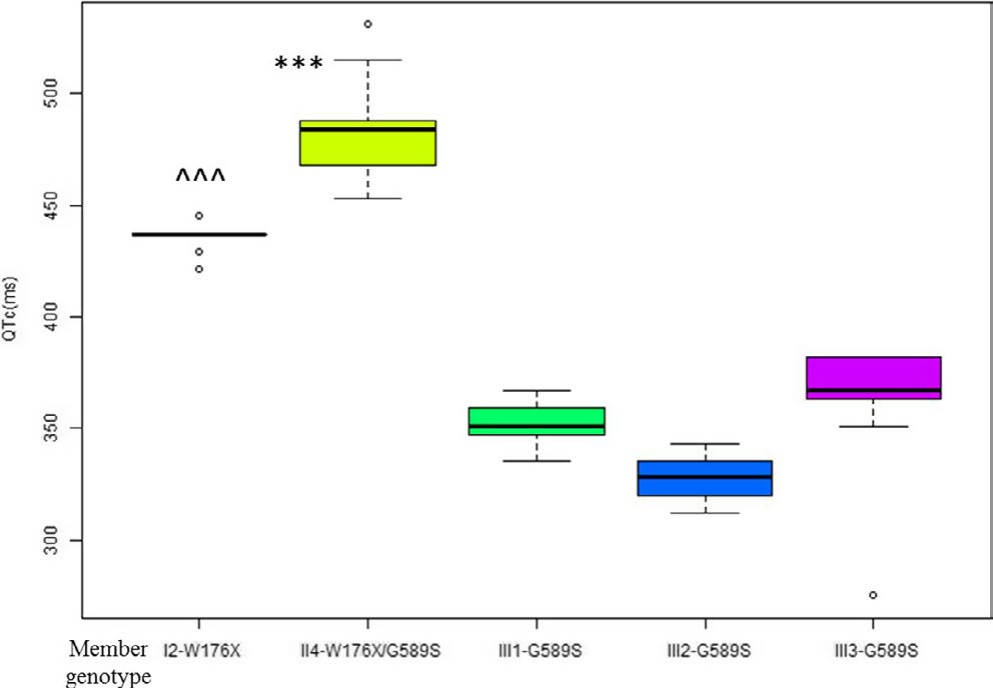
Analysis of the mean QTc interval/per hour in the Holter recording. Note: The QTc here was defined as the mean QTc interval/per hour in the Holter recording during the resting and exercise states. ms, millisecond. ***p value < 0.001, the QTc of II:4 compared with that of I:2, III:1, III:2, and III:3 members; ^^^*p* value < 0.001, the QTc of I:2 compared with III:1, III:2, and III:3

The Holter recordings (The Remote Electrocardiogram Monitoring Center of Family Doctors in Guangdong Province and CIM TECHNOLOGY INC, in CHINA) were conducted for the I:2, II:4, III:1, III:2, and III:3 members during the resting and exercise states. The II:1 and II:5 members refused the Holter recording. Continuous data were expressed as mean ± *SD* and analyzed by the ANOVA method. All statistical analyses were performed using Empowerstata software. *p* values < .05 indicated statistical significance. The values of mean QTc/per hour in the Holter recording were as follows: I:2, 435.86 ± 6.16 ms; II:4, 483.54 ± 21.30 ms; III:1, 352.67 ± 8.17 ms; III:2, 329.25 ± 9.30 ms; and III:3, 366.17 ± 21.88 ms (Figure 4). For II:4, there were dynamic prolongation of QT interval, transient sinus arrest and subsequent ventricular pacing during the sleeping state. Due to fear of recurrence of harmful symptom listed above, she refused to participate into or create any inducing factors and environment. There was no cardiac event during the recordings of I:2, III:1, III:2, and III:3. The 12‐lead ECGs of all three single G589S mutation carriers were shown in Figure S1.

## 5 IN‐SILICO ANALYSIS

In‐silico analysis was used to evaluate the changes of the RNA‐protein secondary structure and the protein physics‐chemical parameters (Qureshi et al., 2013).

### 5.1 RNA secondary structure prediction

The RNA secondary structure was predicted by RNAfold WebSever. The difference of the minimum free energy (MFE) in the centroid secondary structure was evaluated between the mutant mRNA and wild mRNA. The MFE of c.G527A mutation (−585.36 kcal/mol) was much lower than that of the wild type (−359.16 kcal/mol), which thus lead to an improvement of the structural stability. Whereas the MFE of c.G1765A mutation (−333.70 kcal/mol) was approximately similar to that of the wild type, which therefore probably induced no obvious change in the centroid secondary structure (Table 3).

**Table 3:** RNA secondary structure prediction

Mutation	MEF of the centroid secondary structure (kcal/mol)	Image of the centroid secondary structure
WILD	−359.16	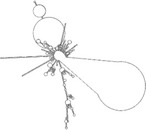
W176X	−585.36	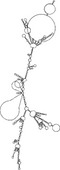
G589S	−333.70	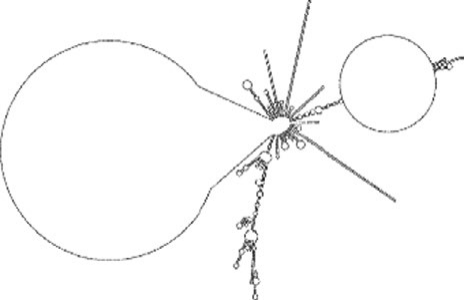

### 5.2 Protein secondary structure prediction

**Figure 5:**
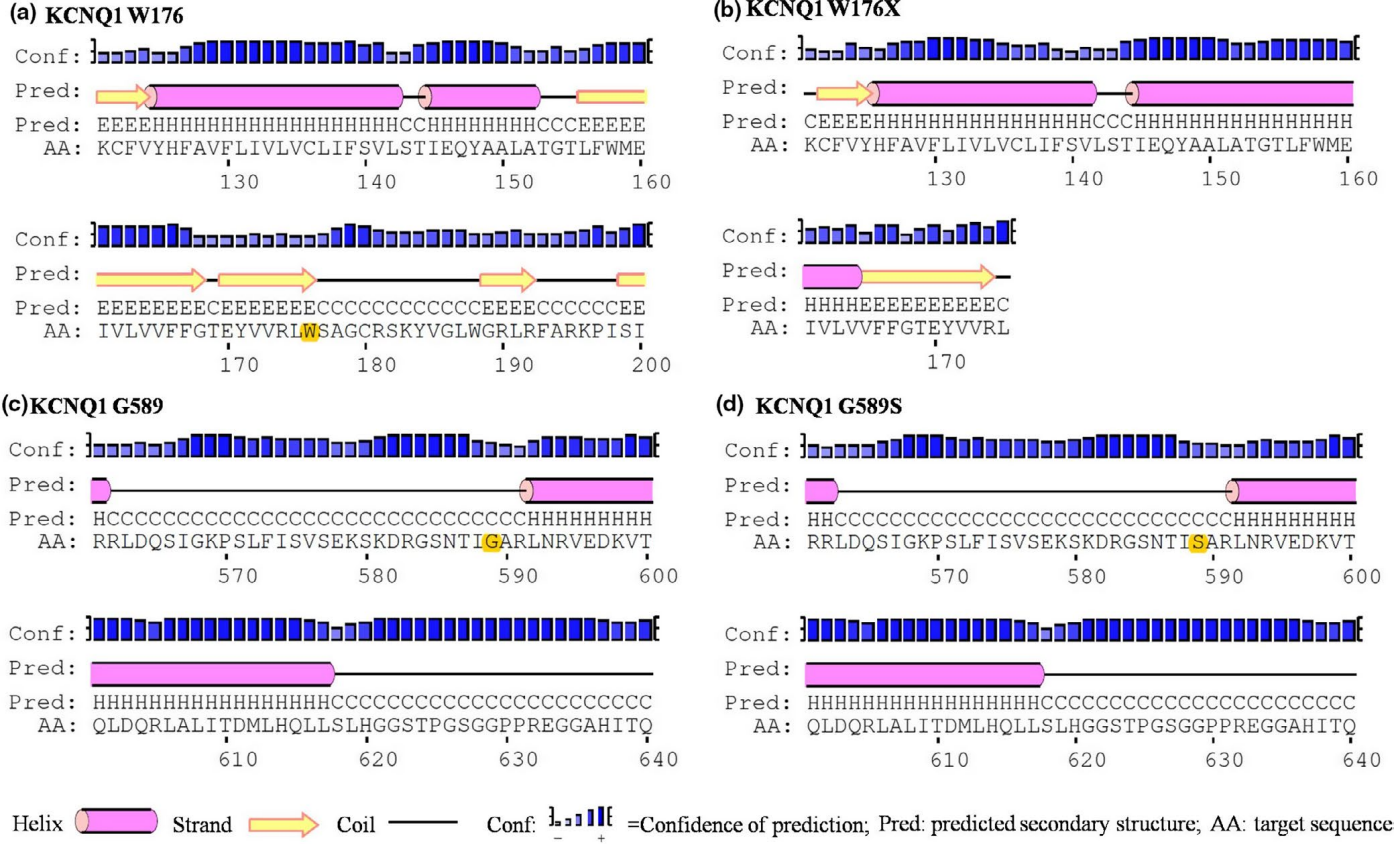
The Changes of protein secondary structure predicted by Phyre2 software. Note: A: secondary structure nears the W176 residue in protein of wild‐type KCNQ1. B: secondary structure nears the W176 residue in protein of W176X mutation of KCNQ1. C: secondary structure nears the G589 residue in protein of wild‐type KCNQ1. D: secondary structure nears the G589 residue in protein of G589S mutation of KCNQ1

As evaluated by the Phyre2 software, the protein secondary structure of KCNQ1 p.W176X was completely lost since the 175th position and the secondary structure before the 175th position also showed significant changes (Figure5a,b). In addition, the protein secondary structure of G589S was only slightly shortened in its previous helix structure compared with the wild type (Figure5c,d).

### 5.3 Protein physics and chemical parameters prediction

Predicted by the ProtParam tool, it can be seen that compared with the wild type, the Theoretical pI, Instability index, Aliphatic index, and Grand average of hydropathicity (GRAVY) of W176X showed an increasing trend. The Instability index of G589S also showed a slight increase (Table 4).

**Table 4:** Protein physics and chemical parameters prediction

Physics and chemical parameters	WILD	W176X	G589S
Molecular weight	74,698.60	18,699.90	74,728.62
Theoretical pI	9.88	10.02	9.88
Instability index	41.24	59.29	41.37
Aliphatic index	90.28	92.06	90.28
Grand average of hydropathicity (GRAVY)	−0.093	0.275	−0.094
Number of amino acids	676	175	676
Total number of negatively charged residues (Asp + Glu)	53	9	53
Total number of positively charged residues (Arg + Lys)	86	18	86

## 6 DISCUSSION

In this Chinese family, the coexisting compound heterozygous mutations of W176X and G589S in KCNQ1 gene may be the potentially pathogenic and genetic risks for the proband of type 1 LQTS with a familial history of USD.

### 6.1 KCNQ1 gene and long QT syndrome

KCNQ1 gene is located on the chromosome 11p15.5‐p15.4, containing 16 exons and encompassing ~400 kb (Lee, Hu, Johnson, & Feinberg, 1997). KCNQ1 encodes an α subunit of the voltage‐gated K + channel KvLQT1 (KV7.1), which has six transmembrane domains (S1–S6), one pore loop, and intracellular NH_2_ and COOH terminals (Wang et al., 1996; Yang et al., 1997). The amino acid residues in C‐terminal form approximately 50% of the entire subunit, including four helical regions (helices A‐D) (Haitin & Attali, 2008). KCNQ1 protein is in the form of tetramer as an intact functional channel. KCNQ1 channel plays an important role in numerous tissues, including the heart, inner ear, stomach, and colon. It mediates the slowly activating potassium current (*I*_ks_) characterized by outward rectification, which contributes to repolarization in cells of atria and ventricles (Kinoshita et al., 2014; Schroeder et al., 2000). Moreover, the regulation of the voltage‐gated K + channel can be mainly divided into two important parts: On one hand, as an ion channel, the functional structure of KCNQ1 lies in the transmembrane domains of S1–S6. The voltage sensor domain (VSD) works for voltage‐dependent movements comprising of transmembrane helices S1–S4, and the pore‐gate domain (PGD) works for activation gate movements comprising S5–S6. The interaction between VSD and PGD makes the movements feasible (Cui, 2016). In addition, KCNE1 also interacts with VSD, especially the domain S4 and alters the VSD movement drastically (Nakajo & Kubo, 2015). On the other hand, helices A‐B in C‐terminal mediate the binding of KCNQ1 and CaM in charge of gating, folding, and membrane trafficking of the channel (Kinoshita et al., 2014). More importantly, helix D provides a leucine zipper (LZ) motif for the interaction with Yotiao (also called A‐kinase anchoring protein 9, AKAP9), which plays a key role in the sympathetic nervous system (SNS) regulation of cardiac action potential duration (APD). This means exercise or emotional fluctuation can trigger cardiac events if mutations in this domain lead to severe damage (Jespersen, Grunnet, & Olesen, 2005). Mutations in KCNQ1 causing loss‐of‐function and gain‐of‐function can lead to type 1 LQTS, AF, and even fatal arrhythmia when calcium channels are reactivated causing early afterdepolarizations (Chen, Xu, & Bendahhou, 2003; Keating & Sanguinetti, 2001).

### 6.2 Compound heterozygous mutations associated with long QT syndrome

Some limited reports revealed that compound heterozygous mutations in KCNQ1 genes aggravated the phenotype of LQTS. For example, T391I/Q530X, V310I/R594Q, G314S/P448R, and K318N/V307sp caused the familial LQTS (Westenskow, Splawski, Timothy, Keating, & Sanguinetti, 2004); whereas, G269D/Y171X, G585delfs/D202N, and R518X/Q530X induced the familial JLNS (Ning et al., 2003; Wang et al., 2002). The stop‐codon mutation of W176X was previously reported in clinical genetic testing performed by other laboratories. Nevertheless, it was first identified in the pathogenic individual in this study. The W176X results in a premature stop codon at the position 176th and synthesis of a truncated protein, which means the subunit coded by W176X will lose the significant structure including S3–S6 and helices A‐D. Similarly, the functions associated with these structures are almost lost, such as the normal membrane trafficking, voltage sensing effect of which the S4 domain is the central part (Catterall, 2010), SNS regulation, and more. In‐silico analysis demonstrated W176X in RNA, protein secondary structure, and protein physics‐chemical parameters prediction distinctly altered the normal role in the gene. In addition, I:2 who carried W176X had a longer QTc than III:1, III:2, and III:3 members who carried G589S, which probably means W176X can cause more damage to the *I*_ks_ channel. Interestingly, the Q530X had caused complete loss of *I*_ks_ channel function according to previous research (Westenskow et al., 2004). Therefore, W176X as a truncated mutation also causing complete loss of *I*_ks_ function is one of the important pathogenic factors in this patient.

The mutation G589S, located in the helix D domain of the channel subunit, was first identified in our study. Two mutations G589D and A590T, which were nearby and have similar significance to G589S, were previously reported (Lupoglazoff, Denjoy, & Villain, 2004; Marx, Kurokawa, & Reiken, 2002). As mentioned above, *β*‐adrenergic receptor activation of SNS mediates an increase in cAMP leading PKA stimulation and thereby PKA interacts with KCNQ1 through Yotiao causing the phosphorylation of residue S27. It is believed that the blocking between KCNQ1 and Yotiao by mutation G589D (G589 is the first “e” position in the LZ motif) may lead to the turbulence of SNS modulation which shortens APD and increases the risk of cardiac events such as USD (Marx et al., 2002). Thus, G589S which is the same position as G589D may cause the alike consequence. On the other hand, functional analyses showed that A590T causes a reduction in *I*_ks_ density and voltage‐sensitivity, which can prolong the QT interval. Mutation G589D has also been reported to cause a similar effect on voltage‐sensitivity (Marx et al., 2002). Besides, immunocytochemical and immunoblot analyses demonstrated A590T reduced cell surface expression. These findings suggest that A590 residue, even the near residues, has an important effect on the maintenance of channel surface expression and function (Kinoshita et al., 2014). This mechanism may be another potential pathogenic reason for the G589S.

In this Chinese family, II:4 carried the compound heterozygous mutations of W176X and G589S in KCNQ1 gene from homologous chromosomes. She repeatedly suffered from cardiac syncope triggered by externally, stressful, mental‐psychological stimulation. In the Holter recording after valid ICD therapy, the mean QTc of II:4 was still obviously longer than that of I:2 carrying the W176X and III:1–3 carrying the G589S. This suggests that the compound heterozygous of W176X and G589S lead to more harmful dysfunctions to the I_ks_ channel. Her young sister died of USD when she was running after the bus, which was in accordance with the complication of LQTS. This suggests that her sister may at least carry the same genotype as II:4, and their clinical symptom induced by the stress stimulation was accordant with the manifestation of both mutations affecting the SNS modulation. It was worth mentioning that their parents and relatives who only carried a single mutation had never experienced any clinical symptoms or cardiac events and showed no significant clinical phenotype. We speculated that the single mutation is not enough to cause a disease due to the compensation of another normal chromosome. However, the combination of W176X and G589S in KCNQ1 resulted in a more severe phenotype of syncope, more prolonged QTc interval and even USD. Therefore, the coexisting and interaction of W176X and G589S of KCNQ1 gene served as a recessive inheritance with a compound heterozygous trait were the most important pathogenic and genetic risks for type 1 LQTS and USD in this family. It was still necessary to study the effects of both mutations on the potassium channel function at the cellular level. Furthermore, no adverse cardiac event was evaluated by ICD monitoring after being instructed to avoid inducing factors and conduct stress management. Stress management, especially among symptomatic LQTS mutations carriers might decrease the risk of an adverse cardiac event (Hintsa et al., 2013; Määttänen et al., 2013), and thus stress management should be a key link in the treatment of LQTS.

## 7 CONCLUSIONS

The compound heterozygous mutations of W176X and G589S in KCNQ1 gene from homologous chromosomes were identified in the patient with type 1 LQTS and familial history of USD. Compared with the wild‐type KCNQ1, both mutations significantly changed the RNA and protein secondary structure, protein physics‐chemical parameters predicted by bioinformation algorithms. The coexisting interaction of both mutations, resulting in more severe phenotype, may be the important pathogenic and genetic risks for LQTS and USD.

## CONFLICT OF INTERESTS

The authors declare that they have no competing interests.

## AUTHORS' CONTRIBUTIONS

XL and YL conceived and designed the study. YL and TZ investigated relevant family information, analyzed, and interpreted the patient data regarding the cardiac disease, and wrote the article. SH, JH, and QL organized the data and produced the figures and tables. NY performed the experiment and provided the mutants. All authors read and approved the final manuscript.

## ETHICS APPROVAL AND CONSENT TO PARTICIPATE

This study was approved by the Guangdong Medical Institutional Review Board and Medical Ethics Committees [No.GDREC2016001H (R1)]. All participants gave informed consent.

## CONSENT FOR PUBLICATION

The written informed consent or parental consent for publication was obtained from all participants.

## Data Availability

The dataset supporting the conclusions of this article are included within the article [and its Additional file(s)].
