# Supplementary figures and images for: Compound and heterozygous mutations of KCNQ1 in long QT syndrome with familial history of unexplained sudden death: Identified by analysis of whole exome sequencing and predisposing genes
Source: Ann Noninvasive Electrocardiol. 2019 Sep 29;25(1):e12694. doi: 10.1111/anec.12694 (PMC7358849; doi:10.1111/anec.12694)

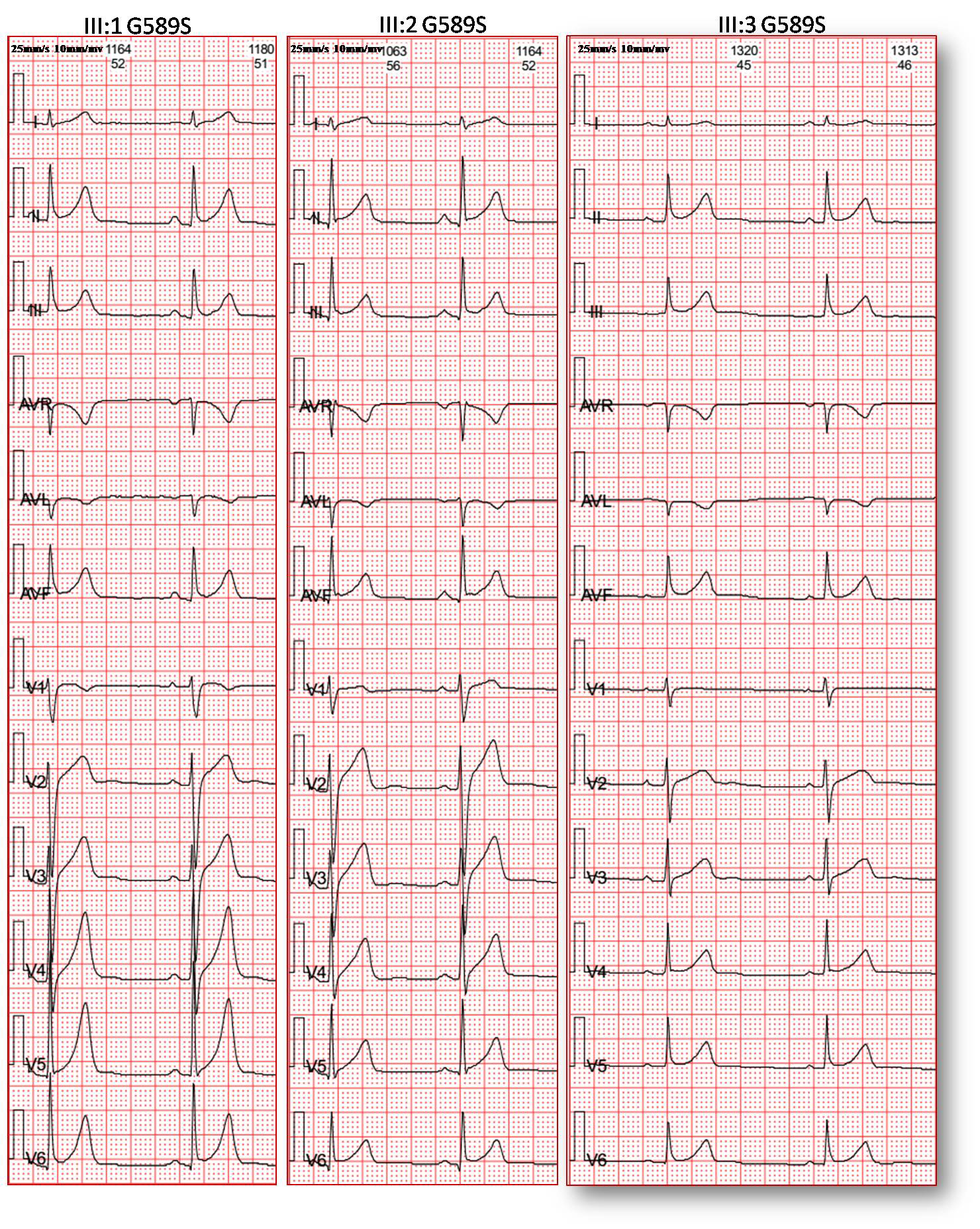

Supplement: Supplementary file 1 [file ANEC-25-e12694-s001.tif]
